# Evaluation of the learning curve for thulium laser enucleation of the prostate with the aid of a simulator tool but without tutoring: comparison of two surgeons with different levels of endoscopic experience

**DOI:** 10.1186/s12894-015-0045-2

**Published:** 2015-06-09

**Authors:** Giovanni Saredi, Giacomo Maria Pirola, Andrea Pacchetti, Jon Alexander Lovisolo, Giacomo Borroni, Federico Sembenini, Alberto Mario Marconi

**Affiliations:** Department of Urology, Ospedale di Circolo e Fondazione Macchi, Varese, Italy; Department of Urology, University of Modena e Reggio Emilia, viale Borri, 57, 21100 Varese, Modena Italy; Department of Urology, Ospedale di Circolo di Busto Arsizio, Saronno, Italy; Department of Surgery, Ospedale di Circolo e Fondazione Macchi, Varese, Italy; Department of Statistics, Bicocca University, Milan, Italy

**Keywords:** Benign prostatic hyperplasia, Endourology, Laser surgery, Prostate disease, Simulator

## Abstract

**Background:**

The aim of this study was to determine the learning curve for thulium laser enucleation of the prostate (ThuLEP) for two surgeons with different levels of urological endoscopic experience.

**Methods:**

From June 2012 to August 2013, ThuLEP was performed on 100 patients in our institution. We present the results of a prospective evaluation during which we analyzed data related to the learning curves for two surgeons of different levels of experience.

**Results:**

The prostatic adenoma volumes ranged from 30 to 130 mL (average 61.2 mL). Surgeons A and B performed 48 and 52 operations, respectively. Six months after surgery, all patients were evaluated with the International Prostate Symptom Score questionnaire, uroflowmetry, and prostate-specific antigen test. Introduced in 2010, ThuLEP consists of blunt enucleation of the prostatic apex and lobes using the sheath of the resectoscope. This maneuver allows clearer visualization of the enucleation plane and precise identification of the prostatic capsule. These conditions permit total resection of the prostatic adenoma and coagulation of small penetrating vessels, thereby reducing the laser emission time. Most of the complications in this series were encountered during morcellation, which in some cases was performed under poor vision because of venous bleeding due to surgical perforation of the capsule during enucleation.

**Conclusions:**

Based on this analysis, we concluded that it is feasible for laser-naive urologists with endoscopic experience to learn to perform ThuLEP without tutoring. Those statements still require further validation in larger multicentric study cohort by several surgeon. The main novelty during the learning process was the use of a simulator that faithfully reproduced all of the surgical steps in prostates of various shapes and volumes.

## Background

Since 1998, holmium laser enucleation of the prostate (HoLEP) has been increasingly used as an alternative to the classic transurethral endoscopic resection of the prostate (TURP) and open prostatectomy [1]. HoLEP is currently considered to be at least equivalent to, or better than, TURP [2]. It shortens the catheterization time and the overall hospital stay. Its functional outcomes are reported to have the same long-term record as open prostatectomy. It has thus been proposed as an endourological procedure that could replace open prostatectomy [3].

Thulium laser, which was introduced in 2005, was found to offer all the advantages of HoLEP for treating bladder outlet obstruction (BOO). The difference between the holmium and thulium beams lies in the latter’s continuous wave mode [4] and tissue penetration of 0.1–0.2 mm compared with the 0.3– to 0.4-mm penetration of holmium [5]. Thulium-YAG laser has been applied during various procedures, such as prostate vaporization (ThuVAP), vaporesection (ThuVARP), vapoenucleation (ThuVEP), and more recently thulium laser enucleation of the prostate (ThuLEP) [6].

ThuLEP was introduced as a minimally invasive, size-independent treatment for BOO using an approach comparable to that for HoLEP [4, 7]. When compared with a holmium laser, thulium seems to deliver improved vaporization ability, ensuring smooth tissue incisions. Because this plane is now more easily distinguishable with ThuLEP, the surgeon is able to remove the adenoma accurately at the level of the surgical capsule. Virtually any prostate size can be removed transurethrally using this technique [8].

For HoLEP, it has been shown that at least 50 cases must be undertaken to achieve a safe learning curve [9]. To date, there are no such data for ThuLEP. Hence, in 2012, two laser-naive surgeons with consolidated experience in traditional urological endoscopy embarked on a pathway to perform this innovative technique after visiting several centers where urological laser surgery was regularly performed.

The aim of this study was to compare retrospectively the learning curves of these two surgeons. Each was an experienced urological surgeon: one had more than 25 years of experience in endoscopic urological surgery (surgeon A), and the other had about 15 years of practice (surgeon B). Both surgeons practiced the steps involved in ThuLEP on a dedicated simulator before performing the procedure on humans but had no formal tutoring.

## Methods

Between June 2012 and August 2013, a total of 100 patients underwent ThuLEP at our institution. Two laser-naive surgeons (A and B) who had long-term experience in traditional urological endoscopy performed all of the ThuLEP procedures.

A novelty in this case series was the availability of a new tool (CyberSim; Quanta System, Solbiate Olona VA, Italy). This simulation instrument clearly reproduces the various steps of the operation [10, 11]. The simulator is able to reproduce prostatic adenomas of different volumes and shapes, different lengths of the prostatic urethra, and particularly different median lobe morphologies. After each simulated procedure, the system gives a report on the resection rate (with preoperative and postoperative views) and shows the areas of residual adenoma. It also reports errors, such as surgical capsule perforation, and the presence of striated sphincter lesions. To test various modalities of coagulation, it is possible to induce “bleeding” during the procedure. Each of the two surgeons was offered the possibility of practicing for a 2-week period.

Prior to surgery all patients underwent clinical evaluation including assessment of the International Prostate Symptom Score (IPSS) questionnaire, uroflowmetry, post-void residual urine evaluation, and transrectal ultrasonography (TRUS). Patients with prostatic adenoma volumes of 30–150 mL were considered candidates for ThuLEP, whereas those with adenomas <30 mL underwent ThuVAP (150 W). Those with adenomas >150 mL were treated by open simple prostatectomy. Patients with cardiovascular disease (assuming that the patient was under care with anti-platelet drugs) were not excluded, except for one case in which the patient was receiving doubled anti-platelet therapy. Additionally, patients on oral anticoagulant therapy were shifted to low-molecular-weight heparin.

We obtained institutional review board approval for this study. Written informed consent was always obtained from the patient before surgery.

Exclusion criteria were a low IPSS score (<7 points), urodynamic evidence of neurogenic acontractile bladder detrusor, and/or a history of prostate surgery. We recorded the total surgical time, total laser emission time, total delivered laser energy (joules), laser fiber caliber, days of postoperative catheterization with and without continuous saline bladder irrigation, and the length of postoperative hospital stay.

Spinal anesthesia was mainly used. All operations were performed with the same laser machine (Cyber TM 150; Quanta System, Solbiate Olona VA, Italy) using two different end-firing fibers (calibers of 600 and 800 μm, depending on the prostate volume and the surgeon’s preference). A mechanical tissue morcellator (Piranha; Wolf, Knittlingen, Germany) was used in all but five cases, in which a different device (DRILLCUT; Karl Storz, Tuttlingen, Germany) was used. Continuous bladder irrigation was employed for the first 12 h postoperatively in all cases. Indwelling catheters were removed on the first postoperative day if hematuria was not present. The Clavien–Dindo classification was used to assess early and late surgical complications. All patients were followed for at least 6 months postoperatively, at which time functional and subjective outcomes were recorded using the same tests that were applied as preoperative baseline studies (IPSS questionnaire, uroflowmetry, prostate-specific antigen [PSA] test) as well as urinalysis, urine culture, and echography to evaluate the post-void residual volume. Intraoperative data and data regarding clinical outcome were compared between surgeons A and B. Multivariate analysis was used to compare laser emission time (min) and prostatic adenoma volume (mL). We did not consider the total surgical time as it was influenced by variables not directly related to the intervention itself (e.g., instrument changes, technical delays, initial cystoscopy). We also created another variable linked to experience in an attempt to determine if it influenced the laser emission time. We verified our data with the Student’s *t*-test and the Shapiro-Wilk test.

A “stepwise” multiple regression analysis was performed on data from 90 of the participants. Patients who underwent multiple procedures simultaneously—ureterorenoscopy (two cases) and bladder calculus lithotripsy (eight cases)—were excluded. The Ethics Committee of the Faculty of Medicine, University of Insubria, Varese e Como, Italy, approved this study.

### Surgical technique

We employed the classic technique [4] with the Quanta System Cyber TM 150. First, the ureteral orifices were identified, and a coagulation marker (60 W) was placed approximately 1 cm away from each orifice. Then, after identifying the edge of the external urethral sphincter, two mucosal incisions were made (as wide as possible) at the level of the distal third of the verumontanum toward the 12 o’clock position using a thulium laser set at 60 W. Subsequently, the prostatic median lobe was removed starting from a bilateral bladder neck incision that followed the lateral edges of the prostatic median lobe and extended to the verumontanum, where an inverted-U incision was made close to it. The resectoscope was gradually moved under the enucleation edge of the median lobe, and blunt dissection towards 12 o’clock was performed with laser emission of 110 W. In the absence of a median lobe, we performed incisions at 5 and 7 o’clock, creating an artificial median lobe, or a deep incision on the bladder neck at 6 o’clock that extended to the verumontanum, with subsequent lateral shifting of prostatic lobes (110 W).

We next made an anterior incision that extended from 12 o’clock on the bladder neck to the verumontanum. After identifying the “white plane” of the capsule, we mobilized the anterolateral aspect of the adenoma with a bilateral semicircular motion of the resectoscope towards 2 and 10 o’clock, respectively.

The lateral lobes were enucleated separately, beginning with the left lobe. After deepening the apical incision from 6 o’clock to 2 o’clock, the apical edge of the lateral lobes was bluntly exposed by moving the resectoscope and pushing the lobes in the 2 o’clock direction. After identifying the capsular plane (a white plane with small vessels running in parallel fashion), the perforating vessels were coagulated. The entire lateral lobe was then bluntly and progressively released towards the bladder neck. The released lobe was then dissected, joining the anterior and lateral incision at the 2 o’clock position. After complete mobilization of the apex, we completed the blunt disconnection towards the 12 o’clock position. The same procedure was completed on the opposite side. The prostate fragments were pushed into the bladder by continuous irrigation and were totally morcellated at the end of the enucleation.

## Results

The average patient age was 68.8 years (range 52–85 years, median 69 years) (Table 1). The prostatic adenoma volume ranged from 30 to 130 mL (mean 61.2 mL). For surgeon B, the maximum adenoma volume was 105 mL. Surgeon A performed 48 operations, and surgeon B performed 52. Surgical durations were similar: 34–160 min (median 92 min) for surgeon A and 40–127 min (median 86 min) for surgeon B. Laser emission time ranged from 16.0 to 58.4 min (median 28 min) for surgeon A and from 19.0 to 37.1 min (median 24 min) for surgeon B. The total amount of energy delivered was 84,820–386,920 joules (J) (median 165,650 J) for surgeon A and 87,540–358,327 J (median 163,445 J) for surgeon B. Surgeon A used 600 μm of laser fiber in 18 patients (37.5 %) and 800 μm in 30 patients (62.5 %). Surgeon B used the same fibers in 11 patients (21.1 %) and 41 patients (78.9 %), respectively.

**Table 1:** Perioperative data (Varese, 2014)

	Surgeon A	Surgeon B
Cases (n)	48	52
Median age (yrs, range)	71 (50–86)	68 (51–84)
Median Adenoma volume (TRUS, ml, range)	61 (30–130)	60 (30–105)
Median PSA (ng/dl, range)	6.32 (1.2–37.1)	5.98 (0.7–23.6)
I-PSS moderate/high	24/ 17 (7 n.v.)	25/ 17 (10 n.v.)
Median Surgical time (min, range)	92 (34–160)	86 (40–127)
Median Laser emission time (min, range)	28 (16–58,44)	24 (19–37,16)
Median Total energy delivered (J, range)	165,650 (84,820–386,920)	163,445 (87,540–358,327)
600 μm fiber use (no. cases)	18	11
800 μm fiber use (no. cases)	30	41
Intraoperative complications	•1 Bladder perforation	•1 Bladder perforation
	•1 Intraoperative bleeding, with necessity to delay morcellation	
Contemporary procedures	•3 bladder ELT	•2 URS
		•5 bladder ELT
Reoperations	•1 Morcellation on first postoperative day	•1 Hemostatic TUR (first postoperative day)
	•1 TURP for chronic retention (6 months later)	•1 Morcellation of a residual fragment (1 month later)
		•1 Urethrotomy for bulbar urethral stenosis (9 months later)

Surgeon A performed a consensual endolithotripsy of bladder calculi in three patients, and surgeon B did the same in five patients. Surgeon B performed two consensual ureterorenoscopies—one in a patient with urolithiasis and the other in a second patient who was suspected to have a ureteral neoplasm.

Intraoperative complications occurred in two patients treated by surgeon A: a bladder perforation managed with intraoperative monopolar coagulation, prolonged bladder catheterization (5 days) [Clavien–Dindo (C–D) grade 1], and a case of intraoperative bleeding that required a second procedure to complete the morcellation the following day (C–D grade 3b). Surgeon B experienced one case of bladder perforation during morcellation that was identified and managed conservatively (C–D grade 1).

Reoperations were necessary in four other cases. One patient required TURP for chronic retention 6 months later (surgeon A); one required hemostatic TURP for massive postoperative bleeding 24 h after surgery (surgeon B); there was one case of incomplete morcellation that caused urinary retention and required a second look 1 month later (C–D grade 3b) (surgeon B); and there was one case of urethral stenosis 9 months after ThuLEP (surgeon B).

Patients were discharged after urinating spontaneously on the same day on which the bladder catheter was removed. This occurred on the first postoperative day in 75.0 % (*n* = 36) of patients operated on by surgeon A and in 67.3 % (*n* = 35) of patients operated on by surgeon B. The bladder catheter was removed on the second postoperative day in 13 patients (6 for surgeon A, 7 for surgeon B) and on the third postoperative day in 11 patients (5 for surgeon A, 6 for surgeon B). Five patients retained the bladder catheter for more than 3 days: two for 4 days (hematuria, surgeon B) (C–D grade 2), one for 5 days (bladder perforation, surgeon A) (C–D grade 1), and two for a week because of urinary retention after catheter removal in one case (surgeon B) (C–D grade 1) and a renal injury that occurred during ureteroscopy performed in a patient with a solitary kidney (surgeon B) (C–D grade 1).

The 2-month follow-up consisted of clinical evaluation and administration of the IPSS questionnaire. At the 6-month follow-up, we undertook a new clinical evaluation, PSA testing, and uroflowmetry with a sonographic post-void residual determination. Resolution of the symptoms was reflected in a low postoperative IPSS evaluation (<4 points, with a clear patient statement of improved quality of life) and by ecographic assessment of the absence of post-void residual urine, with normal urinary outflow. These data are summarized in Table 2.

**Table 2:** Postoperative and follow-up data (Varese, 2014)

	Surgeon A	Surgeon B
Bladder catheter removal on 1 st	36 patients	35 patients
postoperative day		
Bladder catheter removal on 2 nd	6 patients	7 patients
postoperative day		
Bladder catheter removal on 3 rd	5 patients	6 patients
postoperative day		
Bladder catheter removal after 3	1 patient	4 patients
rd postoperative day	Bladder perforation	2 cases hematuria
		1 Respiratory illness
		1 post URS ARI in patient with solitary kidney
2 months- IPSS symptoms resolution	40 cases (83.3 %)	47 cases (90.38 %)
Median 6 months- PSA (ng/dl)	0.83 (0.20–3.56)	0.91 (0.27–3.75)
6 months- Qmax, ml/s (median)	24.8 (17–31)	24.4 (18–30)
6 months- PVR, ml (Eco)	5.8 (0–22)	7.2 (0–30)

**Fig. 1:**
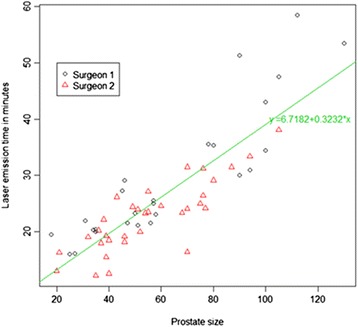
Our Statystical regression model in a scatter plot, showing a linear relation between prostatic adenoma volume (ml) and laser emission time (min). It’s visually clear the absence of statistical differences between the two Surgeons. Varese, 2014

We evaluated our data using a statistical multivariate analysis, noting the laser emission time and prostatic adenoma volume for each surgeon and comparing them. There was a positive linear correlation between these two variables, with a correlation index of 0.856. This model explains the 73 % (R^2^ adjusted = 72.74 %) variability in laser emission time relative to the prostatic adenoma volume. At each 1-mL increment in the prostatic adenoma volume, the laser emission time increased by 0.3232 min. These data were confirmed by the Student’s *t*-test and the Shapiro–Wilk test. All regression coefficients were statistically significant, as shown in Fig. 1.

In the second part of our statistical analysis, we sought a possible relation of the laser emission time (normalized by prostate size) with the amount of urological endoscopic experience. Analyzing the effect of experience (calculated as the number of total operations by an operator) on the laser emission time normalized by prostate size, the linear regression showed that this variable is not statistically significant. The laser emission time did not change significantly during this 100-patient series.

## Discussion

As there is still a lack of studies regarding the learning curve for applying thulium laser surgery to BOO, we tried to compare our data to those obtained using other techniques, such as ThuVEP [12, 13] and HoLEP [8]. HoLEP has been proven to be an effective, minimally invasive, size-independent procedure for the surgical treatment of benign prostatic hyperplasia (BPH). Its prolonged learning curve (requiring specific tutoring for 30–50 cases), however, has prevented this technique from being widely adopted [14].

In 2009, Bach et al. [15] introduced Tm:YAG laser prostatectomy, which initially involved both enucleation of the prostatic lobes and vaporization (Tm-YAG vapoenucleation of the prostate, or ThuVEP). Herrmann et al. [4] introduced the ThuLEP technique in 2010, which involves blunt enucleation of the prostatic apex and lobes using the sheath of the resectoscope. This technique permits easier enucleation because of better visualization and thus the ability to identify precisely the prostatic capsule. This condition, in turn, permits total resection of adenomatous tissue and coagulation of small penetrating vessels. The incidence of postoperative edema and potential complications (e.g., urinary retention, hematuria) is thus reduced [16]. We have refined our technique with the aid of a simulator (Cybersim), which allowed us to practice with “prostatic adenomas” of different sizes and shapes and to focus on the various steps of the operation.

ThuLEP appears to be safe and effective regardless of prostate size, with outcomes comparable to those achieved in case series using HoLEP and ThuVEP [5, 14]. However, there is still a lack of studies concerning the learning curve for mastering the ThuLEP technique. We presented herein the results we obtained for the first 100 consecutive patients who underwent ThuLEP at our institution. The procedures were performed by two surgeons who were laser naive but had 25 years (surgeon A) and 15 years (surgeon B) of experience with traditional urological endoscopy.

Patients with adenoma volumes of 30–50 mL were considered for ThuLEP. Those with adenomas <30 mL were treated by ThuVEP, and those with adenomas >150 mL underwent simple open prostatectomy. As we do not believe that there is an upper size limit for the procedure, our current practice is to employ ThuLEP in all patients with an enlarged prostate.

Our results have shown a low incidence of complications with ThuLEP, comparable to that for HoLEP [14] and ThuVEP [12] and fewer than are seen with classic endoscopic procedures for BPH treatment [16]. The excellent hemostatic properties of thulium laser provides safety for the surgeon, who can avoid important complications even without any tutoring. In this consecutive case series, we faced all types of prostatic adenoma from the beginning, and among the first 100 cases we handled adenoma volumes up to 130 mL. We believe that this demonstrates the safety and efficacy of the method we employed. Our follow-up data confirm complete removal of the adenomatous tissue according to the PSA values, uroflowmetry, sonographic evaluation, and “symptomatic” IPSS score resolution.

Symptomatic evaluation using the IPSS questionnaire 2 months after surgery revealed that 13 patients reported persistence of nocturia at least three times per night. All other respondents reported complete resolution of their symptoms. No patients complained about voiding—only about irritative symptoms (nocturia, pollakiuria). Among those with irritative symptoms, most had had adenomas with volumes of <40 mL, so the symptoms were probably due to the large amount of laser energy delivered. The same evaluation at 6 months showed that only four patents still had those symptoms.

The postoperative outcomes of this study appear comparable to those of other case series in the literature. As all surgical complications were verified within the first 60 cases, we might say that it was sufficient experience to perform the operation well and safely after about 30 cases. All complications were managed successfully, and of the five patients who required reoperation (two for surgeon A, three for surgeon B) only three could be viewed as complications caused by inexperience. Most of the complications in our series were observed during morcellation, and the biggest problem we had was related to poor vision as a result of venous bleeding caused by perforation of the prostatic capsule during enucleation. Obviously, the prior endourological experience of the two operators was beneficial for their rapid learning of the technique. The absence of noticeable differences in the perioperative data and urodynamic results for the patients treated by the two surgeons shows that this technique is feasible even without direct tutoring.

Our results are comparable to those published by high-volume centers, where the technique was performed by various surgeons [12]. Our study has shown that a learning curve of about 30 cases is sufficient for performing this technique without difficulty. This number of cases clearly provides less experience than is needed for HoLEP, which requires 30–50 cases [9].

Our statistical analysis showed a linear correlation between the laser emission time and the prostatic adenoma volume. We did not find any statistically significant influence on the laser emission times by each of the two surgeons’ experience from the beginning to the end of the study period, which could be interpreted to mean that more cases are needed to show this parameter. Furthermore, a possible bias of this work is the fact that we considered the learning curves of only two surgeons, both with a previous consolidated endoscopic urology experience, without being able to demonstrate clear difference between them. Larger multicentric cohort studies of patients operated by several surgeons with different endoscopic experience are still required to provide further validity to Our statements.

## Conclusion

The aim of this study was to evaluate the learning curve for ThuLEP required by two surgeons with different levels of urological endoscopic experience. The learning process began with visits to some centers with experience in HoLEP and ThuLEP. It continued with practicing the ThuLEP procedure with a new simulator (Cybersim). Surgical complications were mostly due to morcellation—not to the enucleation itself—and were easily managed. The results of the study are similar to those from other case studies in the literature for laser treatment of BPH. Our data suggest that the number of cases necessary for an endoscopically experienced urological surgeon to learn ThuLEP is lower than that for HoLEP and that tutoring for this technique is not mandatory. The main novelty in our experience is the long-term use of a new simulator tool that precisely reproduces all the step of this procedure. To the best of our knowledge, simulation of ThuLEP has not been previously described. We found that practicing the procedure in 30 cases is sufficient for a single operator to complete the learning curve. In this 100-patient series, there was no significant reduction in laser emission time from the first cases to the last with the same size adenomas for the two surgeons. The only parameter that showed a linear influence on enucleation time was the adenoma volume. Further validity to those statements will be provided by larger multicentric cohort studies considering the outcomes of several surgeons with different urologic endoscopy experience.

## Abbreviations

ThuLEP: Thulium laser enucleation of the prostate
IPSS: International Prostate Symptoms Score
TRUS: Transrectal ultrasonography
PSA: Prostate-specific antigen
HoLEP: Holmium laser enucleation of the prostate
TURP: Transurethral endoscopic resection of the prostate
BOO: Bladder outlet obstruction
ThuVAP: Thulium prostate vaporization
ThuVARP: Thulium vaporesection of the prostate
ThuVEP: Thulium vapoenucleation of the prostate
PVR: Post-void residual volume
